# Human mesenchymal stem cells derived exosomes inhibit the growth of acute myeloid leukemia cells via regulating miR-23b-5p/TRIM14 pathway

**DOI:** 10.1186/s10020-021-00393-1

**Published:** 2021-10-16

**Authors:** Hui Cheng, Jie Ding, Gusheng Tang, Aijie Huang, Lei Gao, Jianmin Yang, Li Chen

**Affiliations:** Department of Hematology, Changhai, Hospital, Naval Military Medical University, Shanghai, 200433 China

**Keywords:** Exosomes, Human mesenchymal stem cell, Acute myeloid leukemia, miR-23b-5p, TRIM14

## Abstract

**Background:**

Acute myeloid leukemia (AML) is a malignancy commonly seen in adults. Previous studies indicated that TRIM14 played a tumorigenic role in various types of cancer and miR-23b-5p was down-regulated in human mesenchymal stem cell-derived exosomes (HMSC-exos) of AML patients. However, their roles in AML remains unclear. Our study aims to investigate the role of TRIM14 and miR-23b-5p in the pathogenesis of AML.

**Materials and methods:**

The blood specimen was collected from de novo AML patients and healthy donators. Exosomes were extracted from the culture medium of human mesenchymal stem cells under ultracentrifugation. Then exosomes were co-cultured with AML cells to determine the effect of their contents. The cell proliferation was detected by cell counting kit-8 assay, whereas the cell apoptosis was detected by flow cytometry. The expression of miR-23b-5p and TRIM14 was silenced or overexpressed to explore their biological functions in AML. Luciferase reporter assay was conducted to validate the interaction between miR-23b-5p and TRIM14. Gene expression was determined by quantitative real-time PCR and immunoblots.

**Results:**

TRIM14 was significantly increased in AML patients and cell lines. The inhibition of TRIM14 significantly reduced the proliferation and induced the apoptosis of AML cells via activating PI3K/AKT pathway, whereas its overexpression exhibited reversed effects. HMSC-exos could suppress the proliferation of AML cells through the delivery of miR-23b-5p. Moreover, miR-23b-5p inhibited the transcription of TRIM14 by binding on its 3’UTR region. Overexpression of TRIM14 exhibited reversed effect against the function of miR-23b-5p mimic.

**Conclusion:**

TRIM14 could promote the proliferation of AML cells via activating PI3K/AKT pathway, which was reversed by HMSC-exos through delivering miR-23b-5p. These findings indicated that miR-23b-5p and TRIM14 could be applied as potential targets for the treatment of AML.

**Supplementary Information:**

The online version contains supplementary material available at 10.1186/s10020-021-00393-1.

## Introduction

Acute myeloid leukemia (AML) is a malignancy with the prompt growth of immature myeloid cells, which most commonly occurs in older adults and preferentially in males (De Kouchkovsky et al. 2016). Although AML is a relatively rare cancer compared with lung cancer, there are about 20,000 new AML cases each year in the United States and AML accounts for 90% of acute leukemia in adults (Garcia-Manero et al. 2019; Carter et al. 2020). The five-year overall survival rate of AML patients is approximately 25%, whereas patients less than 60 years old had better prognoses compared with those over 60 years old (Dohner et al. 2015). Smoking, exposure to benzene, and previous chemo-radiotherapy are deemed as risk factors of AML. However, the pathogenesis of AML remains to be fully elucidated.

Tripartite motif-containing 14 (TRIM14) is a member of the TRIM family that contains the RING domain with the function of E3 ubiquitin ligase (Feng et al. 2019). TRIM14 was found to be involved in the development of various types of cancers such as gastric cancer, breast cancer, and osteosarcoma (Wang et al.^2018^; Xu et al. 2017; Hu et al. 2019). Previous studies indicated that the other members of the TRIM family such as TRIM31 and TRIM22 were associated with the progression and drug resistance of acute or chronic myeloid leukemia (Xiao et al. 2020; Li et al. 2018). However, the role of TRIM14 in AML remains unknown.

Exosomes are extracellular vesicles produced in most eukaryotic cells containing nucleotides and proteins that can be released and subsequently mediate intercellular interactions (Dhondt et al. 2016). A previous study revealed that exosomes in the bone marrow microenvironment changed the proliferation and migration of hematopoietic progenitor cells, in which messenger RNA (mRNA) and microRNA (miRNA) regulated the biological function and process of these cells (Huan et al. 2013). Moreover, the loss of two miRNAs, miR-145 and miR-146a, in mice could lead to the occurrence of leukemia (Starczynowski et al. 2011). Besides, several miRNAs were demonstrated to correlate with the prognosis of AML patients (Garzon et al. 2008). Therefore, the abnormal expression of microRNAs (miRNAs) is associated with the development of AML. A recent study indicated that miR-23b-5p in human mesenchymal stem cell (HMSC)-derived exosomes (HMSC-exos) in AML patients was significantly decreased (Barrera-Ramirez et al. 2017). Moreover, the inhibition of miR-23b-5p could significantly promote the proliferation and migration of non-small cell lung cancer cells (Hu et al. 2018). However, the regulatory mechanism of miR-23b-5p in the pathogenesis of AML requires further investigation.

The exploration of the expression pattern of miRNA in HMSC-exos can provide a better understanding of the pathogenesis of AML. Therefore, our study was conducted to investigate the role of TRIM14 in the development of AML and explore the regulatory mechanism between miR-23b-5p and TRIM14, aiming to provide novel targets for AML treatment.

## Materials and methods

### Human blood specimens

A total of 25 blood specimens of de novo AML patients or healthy donators were collected in our study. All blood samples were sub-packed in 500 ul sodium citrate (3.2%) tubes, centrifuged, and the plasma snap-frozen in liquid N_2_ and stored at −80 °C for further analysis. All written informed consent was obtained from the patients before the collection. There were 18 male and 7 female enrolled in the present study (average age: 57.2 ± 3.2 years). The blood samples from healthy people (male: 15, female:10; average age: 25.3 ± 2.8 years) were functioned as control. Our study was approved by the Ethics Committee of Changhai Hospital, Naval Military Medical University (Shanghai, China).

### Cell culture

Kasumi-1, HL-60, THP-1, HMSC and normal bone marrow cells were used in this study. All cells were obtained from Cell Bank of Chinese Academy of Sciences. The culture medium was composed of DMEM with 10% fetal bovine serum (FBS) (Gibco, USA). Cells were incubated at 37 °C with 5% CO_2_.

### Isolation and identification of HMSC-exos

HMSC-exos were isolated from health donors according to the methods as previously described (Zhu et al. 2012). The medium was ultracentrifuged at 100,000*g* for 60 min to collect exosomes and 100 KDa MWCO (Millipore) was used for the purification of exosomes. The morphology of HMSC-exos was validated by transmission electron microscopy (TEM). The positive biomarkers (CD44 and CD90) and negative biomarkers (CD34 and CD45) of HMSC (Department of hematology, Changhai Hospital, Shanghai, China) were verified by flow cytometry.

### Fluorescence labeled exosomes and validation of exosome uptake

PKH67 (UR52303, Umibio, China) was used to stain exosomes under the manufacturer’s instruction. A total of 50,000 cells THP-1 cells were inoculated into 24-well plates. The intervention group was incubated with the culture medium which contained PKH67 labeled exosomes. The cell nuclei were stained with DAPI (Vector, CA) and the uptake of exosomes was detected by fluorescence microscopy.

### Extraction of RNA and quantitative real-time PCR

Total RNA of samples was extracted by TRIzol Reagent (Invitrogen, USA) and the cDNA was synthesized. The real-time PCR was conducted by three-step reactions according to the instruction. Gene expression was calculated using the 2^−ΔΔCt^ method. The primers used in this study were listed as follows: miR-23b-5p, F: CGTGGGTTCCTGGCATGC, R: AGTGCAGGGTCCGAGGTATT; U6, F: CTCGCTTCGGCAGCACA, R: AACGCTTCACGAATTTGCGT; TRIM14, F: GGATTTGTGTCTCCGTTCTG, R: TCTGTCTGCCTGGTATTCTG; GAPDH, F: AATCCCATCACCATCTTC, R: AGGCTGTTGTCATACTTC. Three replications were needed for each samples and three independent experiments for each reaction.

### Western blot

Total protein of samples was extracted using RIPA lysis buffer (Beyotime, China). The protein was fractionated on SDS-PAGE and transferred to nitrocellulose membrane (Millipore, USA). The primary antibody was applied at 4 ℃ overnight. The protein was detected by ECL after the application of secondary antibody. All primary antibodies used in this study were list as follows: TRIM14 (15742-1-AP, Proteintech, USA); Cleaved-caspase3 (Ab2302, Abcam, UK); AKT (46191, CST, USA); p-AKT (4060, CST, USA); CD9 (Ab92726, Abcam, UK); CD63 (Ab271286, Abcam, UK); CD81(Ab109201, Abcam, UK); GAPDH (60004-1-1G, Proteintech, USA). Three replications were needed for each samples and three independent experiments for each reaction Cell transfection.

To knockdown the expression of TRIM14, three short interfering RNAs (siRNAs) were generated (Major, China) and constructed into lentiviral plasmids. A negative control siRNA (siNC) was used as the control group. The sequence of three siRNAs were as follows: siTRIM14-1: 5’- GCAGCACATTGACAACATA -3’; siTRIM14-2, 5’- GCCCGTCAAGAGCTTCTTT-3’; siTRIM14-3 5’- GCGATCGCTATTGCTGAAA-3’. For overexpression, a lentiviral plasmid containing TRIM14 cDNA was generated with a mock plasmid as a negative control (oeNC). Lipofectamine 2000 (Invitrogen, USA) facilitated the cell transfection.

### Cell viability assay

Cell proliferation was assessed by cell counting kit-8 (CCK-8) (DOJINDO, Japan). At the time points of 0, 24, 48 and 72 h, cells were co-incubated with CCK-8 solution (1:10) for 1 h. Quantification of cell proliferation was conducted on a microplate reader (Pulangxin, Beijing, P.R. China) with optical densities (ODs) set at a wavelength of 450 nm. Three replications were needed for each samples and three independent experiments for each reaction.

### Flow cytometry

Cells were harvested after 48-h transfection and stained with Annexin V/FITC and PI (BD, USA) following the instruction. The results were analyzed using FACSDiva 7.0 software. Annexin V-FITC^+^ and PI^−^ populations indicated apoptosis. Experiments were conducted in triplicates. Annexin V^−^ and PI^−^ populations were healthy cells that were considered negatively stained. Annexin V^+^ and PI^−^ cells indicated cells in early apoptosis. Moreover, Annexin V^+^ and PI^+^ staining indicated cells in necrosis (postapoptotic necrosis or late apoptosis). Three replications were needed for each samples and three independent experiments for each reaction.

### Dual-luciferase reporter gene assay

Binding sites of miR-23b-5p and TRIM14 were predicted by TargetScan and Starbase. Then wildtype and mutant sequences of TRIM14 were synthesized and cloned to vectors. THP-1 cells were co-transfected with miR-23b-5p mimics or inhibitors. After 48-h transfection, dual-luciferase reporter gene kit was used to determine the luciferase activity. Three replications were needed for each samples and three independent experiments for each reaction.

### Statistical analysis

GraphPad Prism version 7.0 (CA, USA) was used for visualization and analyses. Data were displayed as mean ± standard deviation. Comparisons between two groups were analyzed using Student’s t-test, whereas one-way ANOVA analysis was used to compare the difference in more than two groups. P-value < 0.05 was statistical significance.

## Results

### TRIM14 promoted the proliferation and inhibited the apoptosis of AML

**Fig. 1:**
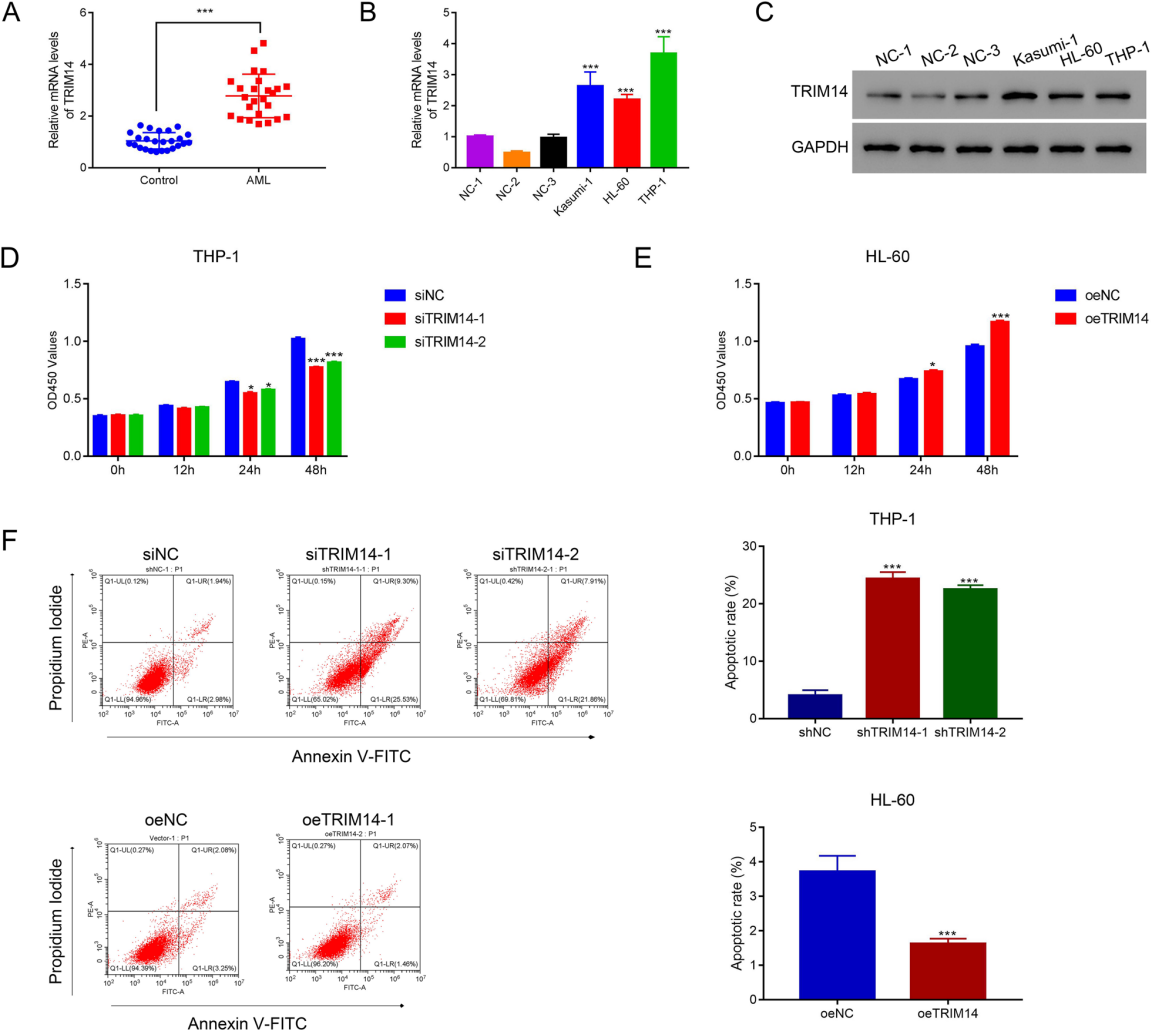
TRIM14 promoted the proliferation and reduced the apoptosis of AML cells. **A** The serum level of TRIM14 in AML patients compared with healthy donators (n = 25 for each group). **B**–**C** The mRNA (**B**) and protein (**C**) levels of TRIM14 in AML cell lines compared with three human normal bone marrow cells 1–3. **D** The proliferation of THP-1 cells after transfecting with siNC, siTRIM14-1 and siTRIM14-2. **E** The proliferation of THP-1 cells after transfecting with TRIM14 overexpressed plasmid. **F** Flow cytometry detected the apoptosis of THP-1 cells after transfecting with siTRIM14-1 and siTRIM14-2. **G** Flow cytometry detected the apoptosis of THP-1 cells after transfecting with TRIM14 overexpressed plasmid and control plasmid. * p < 0.05; *** p < 0.001

To explore the role of TRIM14 in AML, we detected the expression of TRIM14 in the blood from 25 AML patients and healthy donators. Results showed that TRIM14 was significantly elevated in AML patients (p < 0.05) (Fig. 1A). Moreover, TRIM14 was highly expressed in AML cell lines (Kasumi-1, HL-60, and THP-1) compared with normal bone marrow cells (p < 0.05) (Fig. 1B–C). Then we designed three siRNAs and one overexpression plasmid to decrease or elevate the expression of TRIM14. Results indicated that three siRNAs could significantly reduce the expression of TRIM14 (p < 0.05) (Additional file 1: Fig. S1A–B), and TRIM14 was highly expressed after the transfection of overexpression plasmid (p < 0.05) (Additional file 1: Fig. S1C–D). The knockdown of TRIM14 significantly reduced the proliferation of AML cells (p < 0.05), whereas its overexpression exhibited reversed effects (p < 0.05) (Fig. 1D–E). Additionally, the inhibition of TRIM14 significantly increased the apoptosis rate of THP-1 cells (p < 0.05) (Fig. 1F). In contrast, the overexpression of TRIM14 significantly decreased the apoptosis rate of HL-60 cells (p < 0.05) (Fig. 1G). These results indicated that TRIM14 could promote the proliferation and inhibit the apoptosis of AML cells. Importantly, TRIM14 was also induced overexpression using the same lentiviral-mediate overexpression vector in THP-1 cells. CCK8 and flow cytometer were used to examine the proliferation and apoptosis. The similar results were obtained in THP-1 cells (Additional file 2: Fig. S2A–S2C). Our results indicated that the oeTRIM14 was also well functioned in THP-1 cells.

### Inhibition of PI3K/AKT pathway suppressed the function of TRIM14 in AML

**Fig. 2:**
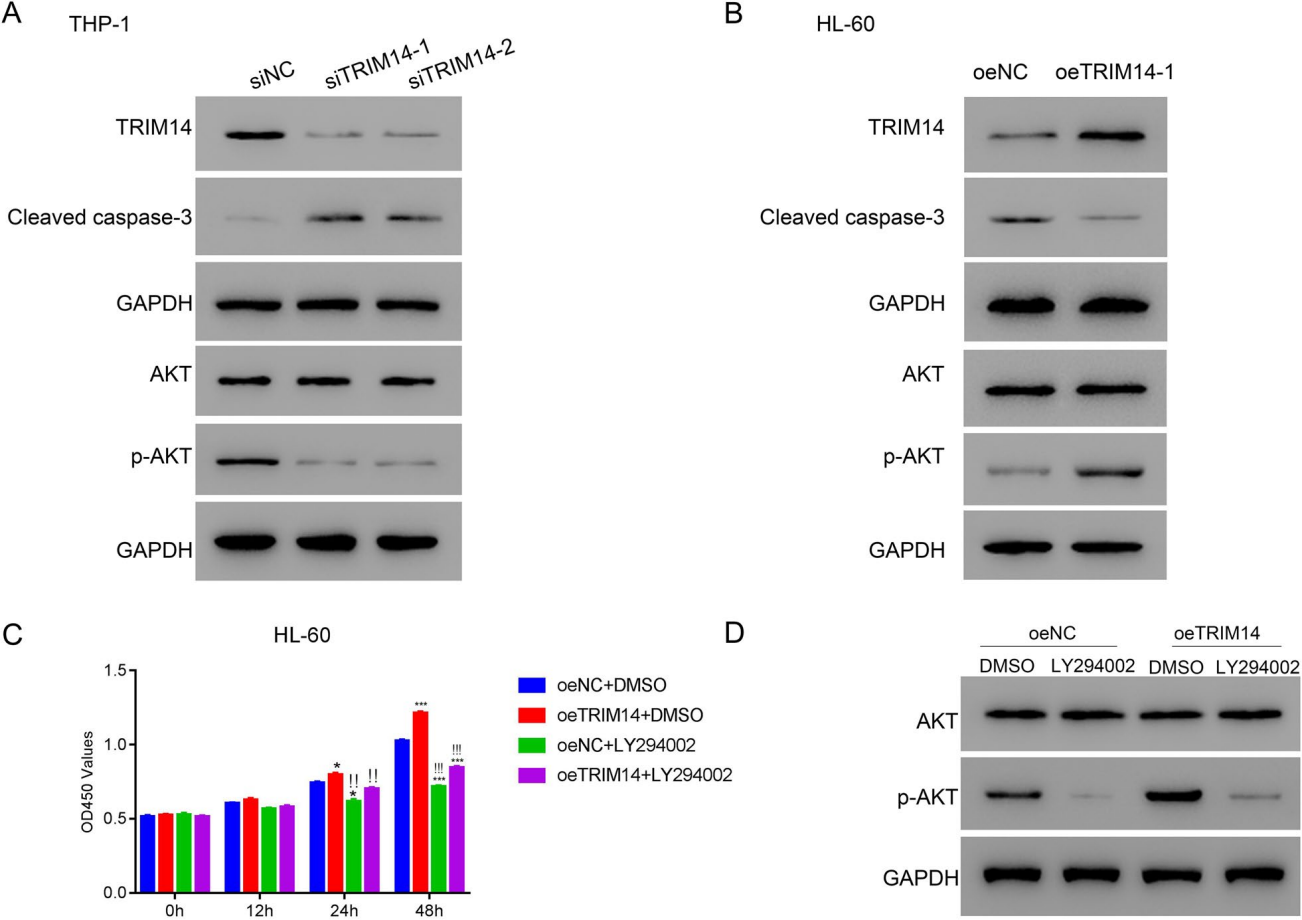
Inhibition of PI3K/AKT pathway suppressed the function of TRIM14 in AML. **A**–**B** The expression of TRIM14, cleaved caspase-3, AKT and p-AKT in AML cells after transfecting with TRIM14 siRNAs (**A**) or overexpressed plasmid (**B**). **C** The proliferation of HL-60 with the application of oeTRIM14 and LY294002. **D** The expression of AKT and p-AKT in HL-60 cells with the application of oeTRIM14 and LY294002. * p < 0.05 vs oeNC + DMSO, *** p < 0.001 vs oeNC + DMSO; !!*p < 0.05 vs oeTRIM14 + DMSO; !!!***p < 0.001 vs oeTRIM14 + DMSO

Previous studies indicated that TRIM14 was associated with PI3K/AKT pathway (Wang et al. 2018; Xu et al. 2017). Therefore, we detected the expression of AKT and cleaved-caspase-3 to explore the underlying mechanism of TRIM14 in AML. In THP-1 cells, the inhibition of TRIM14 markedly elevated the expression of cleaved-caspase-3 and decreased the expression of phosphorylated AKT (p-AKT) and TRIM14 (Fig. 2A). In contrast, the overexpression of TRIM14 notably increased the expression of TRIM14 and p-AKT and decreased the expression of cleaved-caspase-3 (Fig. 2B). Then, the inhibitor of PI3K/AKT pathway, LY294002, was administered to verify the association between TRIM14 and PI3K/AKT pathway. Results showed that the overexpression of TRIM14 significantly promoted the proliferation of HL-60 cells (p < 0.05) (Fig. 2C). However, the application of LY294002 significantly reversed the effect of TRIM14 (p < 0.05) (Fig. 2C). Moreover, LY294002 could markedly inhibit the expression of p-AKT whether TRIM14 was overexpressed or not (Fig. 2D). These results suggested that TRIM14 was associated with PI3K/AKT pathway and the inhibition of PI3K/AKT pathway could suppress the function of TRIM14 in AML.

### HMSC-exos inhibited the proliferation of AML cells by suppressing TRIM14

**Fig. 3:**
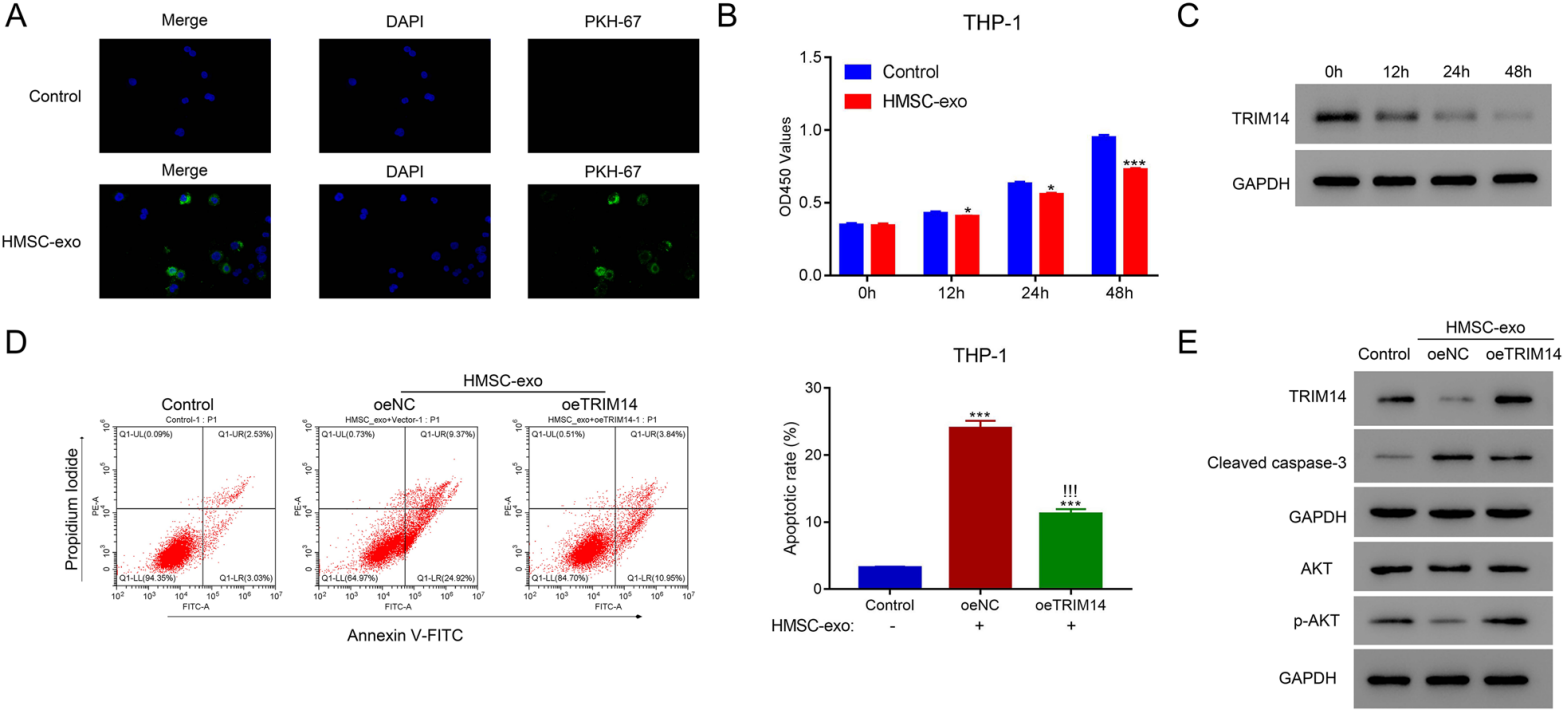
HMSC-exos suppressed the proliferation of AML cells. **A** The uptake of HMSC-exos labeled with PKH-67 by human THP-1 cells was detected using immunofluorescence. **B** The proliferation of THP-1 cells co-cultured with HMSC-exos. **C** The protein level of TRIM14 in THP-1 cells after co-cultured with HMSC-exos. **D** Overexpression of TRIM14 suppressed the apoptosis of THP-1 cells after co-cultured with HMSC-exos. **E** The expression of TRIM14, cleaved caspase-3, AKT and p-AKT in the presence of oeTRIM14 and HMSC-exos. * p < 0.05; *** p < 0.001; !!!*** p < 0.001 vs oeNC

Therapeutics such as stem cells were promising approaches in the treatment of AML. Therefore, HMSC-exos were extracted and co-cultured with AML to explore its potential efficacy. Flow cytometry was conducted to verify the positive (CD44 and CD90) and negative (CD34 and CD45) markers of HMSC (Additional file 3: Fig. S3A). Then, the morphology of HMSC-exos was validated by TEM (Additional file 3: Fig. S3B). Moreover, the expression of CD9, CD63 and CD81 was detected in HMSC-exos (Additional file 3: Fig. S3C). When HMSC-exos were co-cultured with THP-1 cells, HMSC-exos could be absorbed by THP-1 cells and significantly inhibited the proliferation rate (p < 0.05), the control group was designed by THP1 cells without being co-cultured with the HMSC-exos (Fig. 3A–B). Notably, the expression of TRIM14 showed no significant difference in THP-1 at 0, 12, 24 and 48 h without co-cultured with HMSC-exo, while deeply inhibited after the co-culture with HMSC-exos (Fig. 3C). Moreover, the high expression of TRIM14 significantly reduced the apoptosis rate of THP-1 cells after co-cultured with HMSC-exos (p < 0.05) (Fig. 3D). Besides, the overexpression of TRIM14 markedly decreased the level of cleaved-caspase-3 and increased that of p-AKT (Fig. 3E). These findings indicated that HMSC-exos could reduce the proliferation of AML cells by suppressing TRIM14.

### HMSC-exos induced the apoptosis of AML cells by delivering miR-23b-5p

**Fig. 4:**
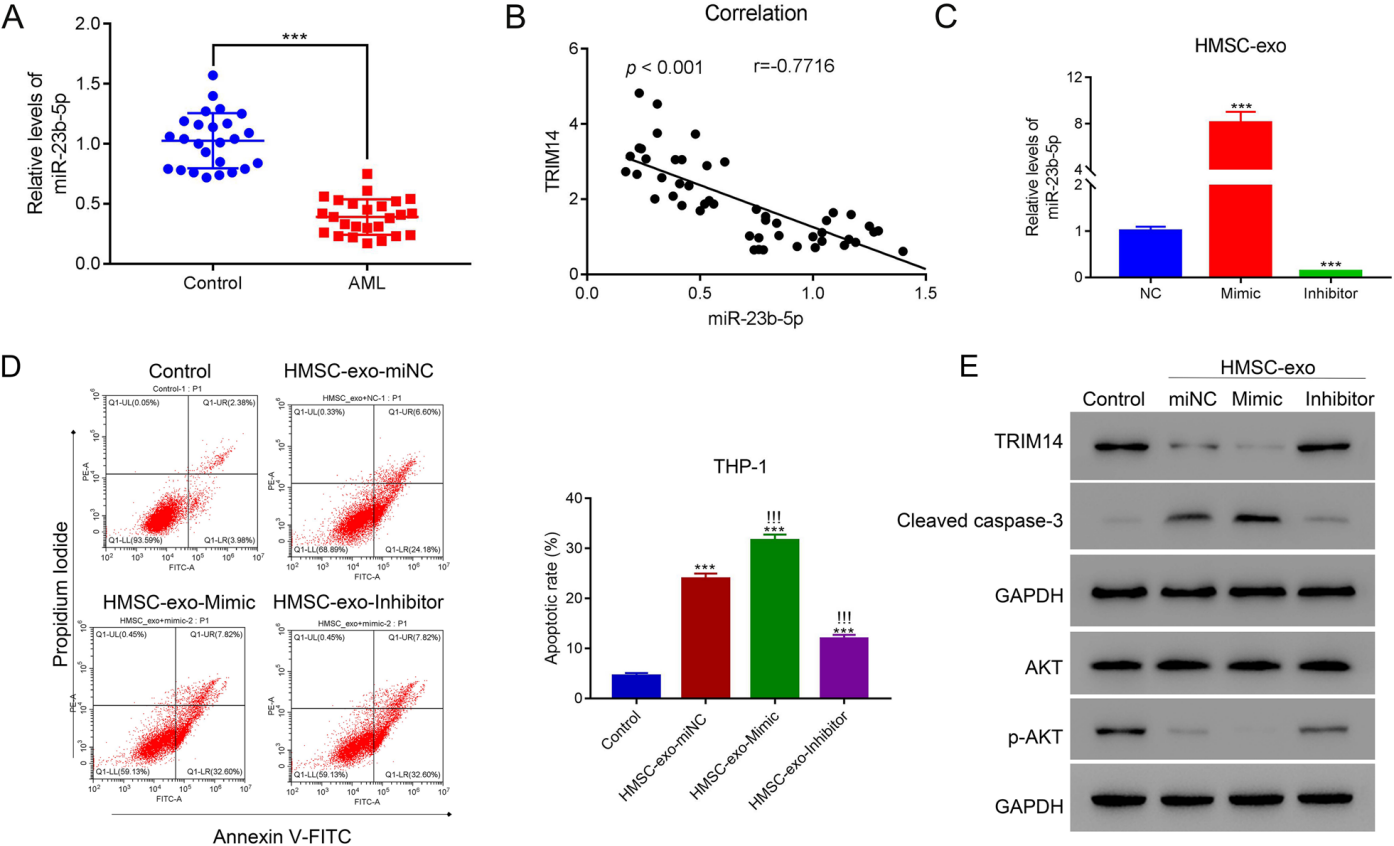
HMSC-exos induced the apoptosis of AML cells through delivering miR-23b-5p. **A** The mRNA level of miR-23b-5p of AML patients and healthy donators (n = 25 for each group). **B** The correlation between miR-23b-5p and TRIM14 in AML patients. **C** The expression of miR-23b-5p in HMSC-exos after co-cultured with miR-23b-5p mimic or inhibitor. **D** Flow cytometry detected the apoptosis of THP-1 cells after co-cultured with HMSC-exo-miNC, HMSC-exo-Mimic and HMSC-exo-inhibitor. **E** The expression of TRIM14, cleaved caspase-3, AKT and p-AKT in THP-1 cells after co-cultured with HMSC-exo-miNC, HMSC-exo-Mimic and HMSC-exo-inhibitor. *** p < 0.001; !!!*** p < 0.001 vs HMSC-exo-miNC

A recent study indicated that miR-23b-5p in HMSC-exos derived from AML patients was significantly reduced (Barrera et al. 2017). Besides, based on the prediction using online databases, TRIM14 was the targeted gene of miR-23b-5p. Therefore, miR-23b-5p might play a crucial role in HMSC-exos in AML. We found that miR-23b-5p expression was significantly declined in AML patients compared with healthy donators (p < 0.05) (Fig. 4A). Besides, TRIM14 was negatively associated with miR-23b-5p in the serum of AML patients (p < 0.05) (Fig. 4B). To explore the biological activities of miR-23b-5p in AML, we designed miR-23b-5p mimic and inhibitor to increase or decrease its expression. The miR-23b-5p mimic could significantly elevate the expression of miR-23b-5p whereas the inhibitor exhibited reversed effects (p < 0.05) (Fig. 4C; Additional file 4: Fig. S4). The overexpression of miR-23b-5p significantly promoted the apoptosis of THP-1 cells whereas its inhibitor significantly reduced their apoptosis rates (p < 0.05) (Fig. 4D). Moreover, miR-23b-5p in HMSC-exos markedly decreased the expression of TRIM14 and p-AKT and increased that of caspase-3 (Fig. 4E). These results indicated that HMSC-exos could suppress the apoptosis of AML cells through delivering miR-23b-5p and miR-23b-5p could inhibit the expression of TRIM14.

### miR-23b-5p inhibited TRIM14 by binding on its 3’UTR region

**Fig. 5:**
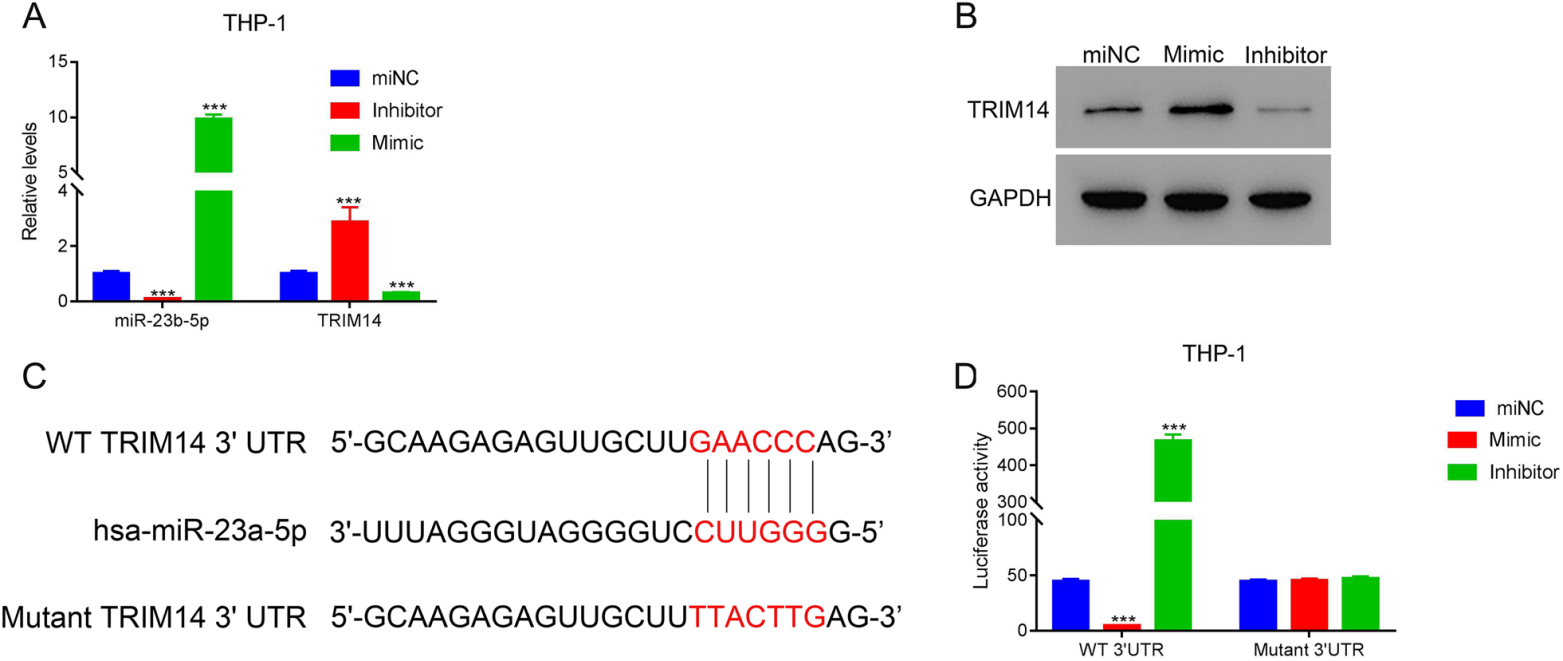
miR-23a-5p suppressed the expression of TRIM14 through binding on its 3’UTR in AML cells. **A** The level of miR-23b-5p and TRIM14 in THP-1 cells after the transfection of miR-23b-5p miNC, inhibitor and mimic. **B** The protein of TRIM14 was in THP-1 cells after the transfection of miR-23b-5p miNC, inhibitor and mimic. **C** The potential binding site of miR-23a-5p in the 3’UTR region of TRIM14. **D** The luciferase activity THP-1 cells after co-transfected with wildtype and mutant TRIM14 vectors as well as miR-23a-5p miNC, mimic and inhibitor. *** p < 0.001

Further, we explored the underlying interaction between miR-23b-5p and TRIM14. Firstly, we transfected the mimic and inhibitor of TRIM14 into THP-1 cells, which induced the up-regulation and down-regulation of miR-23b-5p (p < 0.05) (Fig. 5A). Besides, TRIM14 expression was significantly elevated or reduced after the transfection of miR-23b-5p mimic or inhibitor, respectively (p < 0.05) (Fig. 5A). The alternation of TRIM14 induced by miR-23b-5p mimic and inhibitor was further validated by western blot (Fig. 5B). After predicting the potential binding site of miR-23b-5p and TRIM14, we designed the vectors containing wildtype and mutant sequence of 3’UTR region of TRIM14 and transfected them with miR-23b-5p mimic and inhibitor in THP-1 cells (Fig. 5C). The miR-23b-5p mimic significantly reduced the luciferase activity whereas its inhibitor exhibited the reversed effect (p < 0.05) (Fig. 5D). These results suggested that miR-23b-5p could suppress the expression of TRIM14 by binding on its 3’UTR region.

### Overexpression of TRIM14 reversed the function of miR-23b-5p in AML cells

**Fig. 6:**
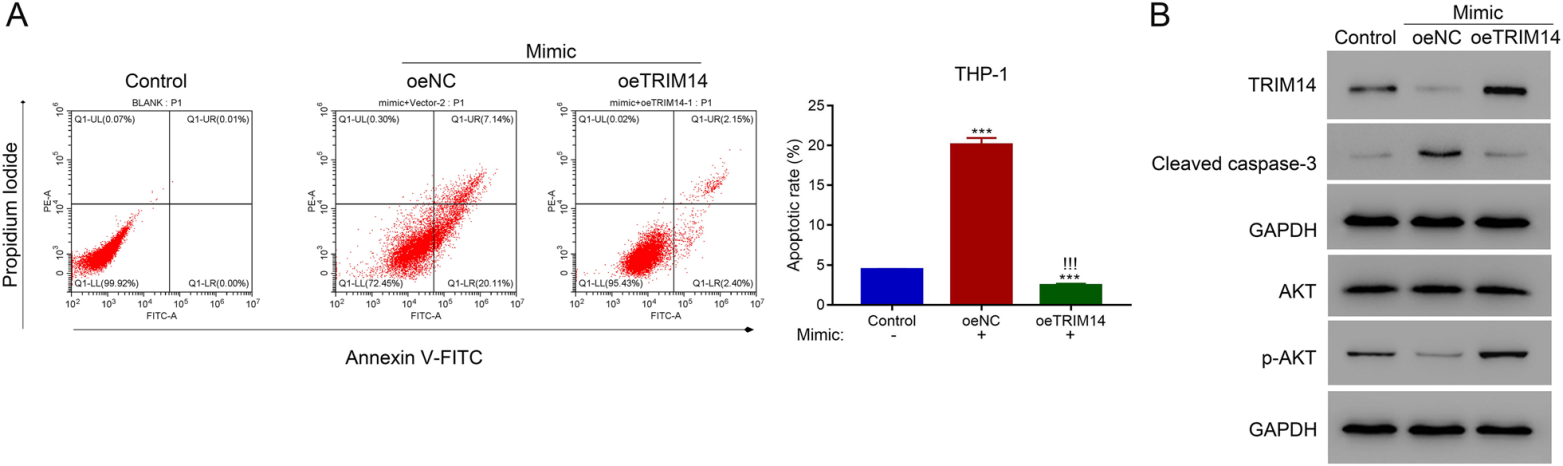
Overexpression of TRIM14 suppressed the function of miR-23a-5p in AML cells. **A** Flow cytometry detected the apoptosis of THP-1 cells after transfecting with TRIM14 overexpressed plasmid and control plasmid in the presence of miR-23a-5p mimic. **B** The expression of TRIM14, cleaved caspase-3, AKT and p-AKT after transfecting with TRIM14 overexpressed plasmid and control plasmid in the presence of miR-23a-5p mimic. *** p < 0.001; !!!*** p < 0.001 vs oeNC + Mimic

To validate the interaction between TRIM14 and miR-23b-5p, we transfected THP-1 cells with TRIM14 overexpression plasmid and miR-23b-5p mimic. Flow cytometry revealed that miR-23b-5p mimic significantly elevated the apoptosis rate of THP-1 cells whereas the overexpression of TRIM14 significantly inhibited the apoptosis (p < 0.05) (Fig. 6A). Moreover, miR-23b-5p mimic markedly increased the level of cleaved caspase-3 and decreased that of p-AKT, which was reversed by the overexpression of TRIM14 (Fig. 6B). These results indicated that miR-23b-5p and TRIM14 played a competent role in regulating the proliferation of AML cells.

## Discussion

Our study revealed that miR-23b-5p/TRIM14 pathway was highly involved in AML. The high expression of TRIM14 in AML patients promoted the proliferation of AML cells via regulating PI3K/AKT pathway. Moreover, HMSC-exos could significantly inhibit the function and expression of TRIM14 through the delivery of miR-23b-5p. These findings suggested that TRIM14 and miR-23b-5p was involved in the progression of AML and provided a novel target for the application of stem cell therapy in AML.

The abnormal proliferation and differentiation of myeloid cells was the main pathology of AML. Chromosomal rearrangements such as chromosomal translocation can interfere with the maturation of myeloid precursor cells (De Kouchkovsky et al. 2016). However, the pathogenesis of AML remains to be fully elucidated. Our study revealed that TRIM14 was highly expressed in AML patients compared with healthy donators, which indicated the potential oncogenic role of TRIM14 in AML. TRIM14 has been implicated to play an oncogenic role in different cancers. Tan et al. revealed that TRIM14 was associated with poor prognosis and chemoresistance by activating Wnt/β-catenin pathway in gliomas (Tan et al. 2018). Moreover, the inhibition of TRIM14 could decrease the proliferation of papillary thyroid carcinoma (Sun et al. 2020). Furthermore, previous studies have reported that signaling pathways such as JAK/STAT and PI3K/AKT pathways are associated with the promoted development of AML (Cook et al. 2014; Nepstad et al. 2020). Our study suggested that TRIM14 could promote the proliferation and reduce the apoptosis of AML cells, which could be suppressed by PI3K/AKT inhibitor, LY294002. Therefore, TRIM14 could promote the progression of AML via regulating PI3K/AKT pathway and could be a potential target for AML treatment.

Conventional treatment for AML consists of chemotherapy, which contains induction therapy and consolidation therapy. The induction therapy was to maximally eliminate tumor cells whereas consolidation therapy is to eradicate residual disease. Besides, stem cell transplantation is another approach for relapsed AML patients who are tolerable to transplant and have a matched donor (Dohner et al. 2015). However, graft-versus-host disease remains a challenge for patients receiving stem cell transplantation. Therefore, there is a clear urgent to develop novel therapeutic strategies for AML. A recent study revealed that the transplantation of stem cell could improve the prognosis of AML patients, which indicated the therapeutic effect of hematopoietic stem cells (Liu et al. 2015). Our study showed that the co-culture of HMSC-exos with AML cells could significantly inhibit the proliferation of AML cells and the expression of TRIM14 in a time-dependent manner. Moreover, the overexpression of TRIM14 could reverse the inhibitory effect of HMSC-exos. Therefore, our study revealed that HMSC-exos exerted therapeutic effect against AML via the inhibition of TRIM14.

Exosomes are carriers of RNA and protein and mediate intercellular communication through the delivery of their contents. A recent study suggested that HMSC-exos could suppress the proliferation of AML cells via the delivery of miR-222-3p to inhibit IRF2 and INPP4B (Zhang et al. 2020). Moreover, miR-23b-5p expression was reduced in HMSC-exos of AML patients, indicating a protective role in AML (Barrera et al. 2017). Besides, TRIM14 was predicted as a potential target of miR-23b-5p. Therefore, miR-23b-5p in HMSC-exos might mediate the inhibitory effect on cell proliferation in AML. Our study found that miR-23b-5p in HMSC-exos could significantly promote the apoptosis of AML cells via the inhibition of TRIM14, and the overexpression of TRIM14 could reverse the inhibitory effect of HMSC-exos. These findings suggested that HMSC-exos exhibited potential therapeutic effects against AML via regulating miR-23b-5p/TRIM14 pathway. Our study revealed the molecular mechanism of HMSC-exos in regulating the proliferation of AML cells and provided a novel approach for the treatment of AML. Notably, it will be valuable to further confirm our findings in AML primary samples. However, this section was not conducted in present research for certain conditions, which limited the significance of present research. Nevertheless, we would like to further validate our results in AML primary samples and in vivo experiment in our following analyses. Age is one of the contributing factors for human blood specimens. In the current study, the age of controls is significantly lower in the controls compared to AML patients. To date, this specific limitation is mainly due to the shortage of patient. Although we tired our best to collect more samples, the age-matched normal and AML blood samples are still not available. Nevertheless, we would like to further validate our results in AML primary samples and in vivo experiment in our following analyses with a larger sample population. Moreover, AML contains many subtypes (Fasan et al. 2014), it will be worth discussing and investigating the role of TRIM14-activated PI3K/AKT pathway in different subtypes of AML.

## Conclusions

To sum up, our study revealed that TRIM14 could promote the proliferation of AML cells via activating PI3K/AKT pathway. HMSC-exos could reverse the oncogenic effect of TRIM14 through delivering miR-23b-5p and exhibited as a potential approach for the treatment of AML.

## Supplementary Information

**Additional file 1: Fig. S1.** The inhibition and overexpression of TRIM14 in THP-1cells. **A**–**B** The mRNA (A) and protein (B) levels of TRIM14 after transfecting with TRIM14 siRNAs in THP-1 cells. **C**–**D** The mRNA (C) and protein (D) levels of TRIM14 after transfecting with TRIM14 overexpressed plasmid in HL-60 cells. *** p < 0.001.

**Additional file 2: Fig. S2.** TRIM14 overexpression promoted the proliferation and inhibited apoptosis in human THP-1 cells. **A**. The proliferation of THP-1 cells after transfecting with oeNC and oeTRIM14 were examined at 0, 12, 24 and 48 h. * p < 0.05 vs oeNC, *** p < 0.001 vs oeNC. **B**. Flow cytometer was used to examine the apoptosis of THP-1 cells after transfecting with oeNC and oeTRIM14. *** p < 0.001 vs oeNC. **C**. Western blot was used to examine the protein levels of TRIM14, AKT, p-AKT, cleaved caspase3 in THP-1 cells after transfecting with oeNC and oeTRIM14.

**Additional file 3: Fig. S3.** Identification of exosomes derived from HMSC. **A**. The positive (CD44 and CD90) and negative (CD34 and CD45) biomarkers for HMSC derived exosomes were detected by flow cytometry. **B**. Transmission electron microscopy revealed the morphology of HMSC-exos (scale bar = 100 nm). **C**. The expression of exosomal biomarkers (CD9, CD63 and CD81) were positive in HMSC-exos.

**Additional file 4: ** Fig. S4. The expression of miR-23b-5p after the application of miR-23b-5p mimic and inhibitor. *** p < 0.001.

## Data Availability

The dataset used and/or analyzed during the current study are available from the corresponding author on reasonable request.

## Abbreviations

AML: Acute myeloid leukemia
HMSC-exos: Human mesenchymal stem cell-derived exosomes
TRIM14: Tripartite motif-containing 14
HMSC: Human mesenchymal stem cell
FBS: Fetal bovine serum
TEM: Transmission electron microscopy
CCK-8: Cell counting kit-8
