# Supplementary figures and images for: Human mesenchymal stem cells derived exosomes inhibit the growth of acute myeloid leukemia cells via regulating miR-23b-5p/TRIM14 pathway
Source: Mol Med. 2021 Oct 16;27:128. doi: 10.1186/s10020-021-00393-1 (PMC8520262; doi:10.1186/s10020-021-00393-1)

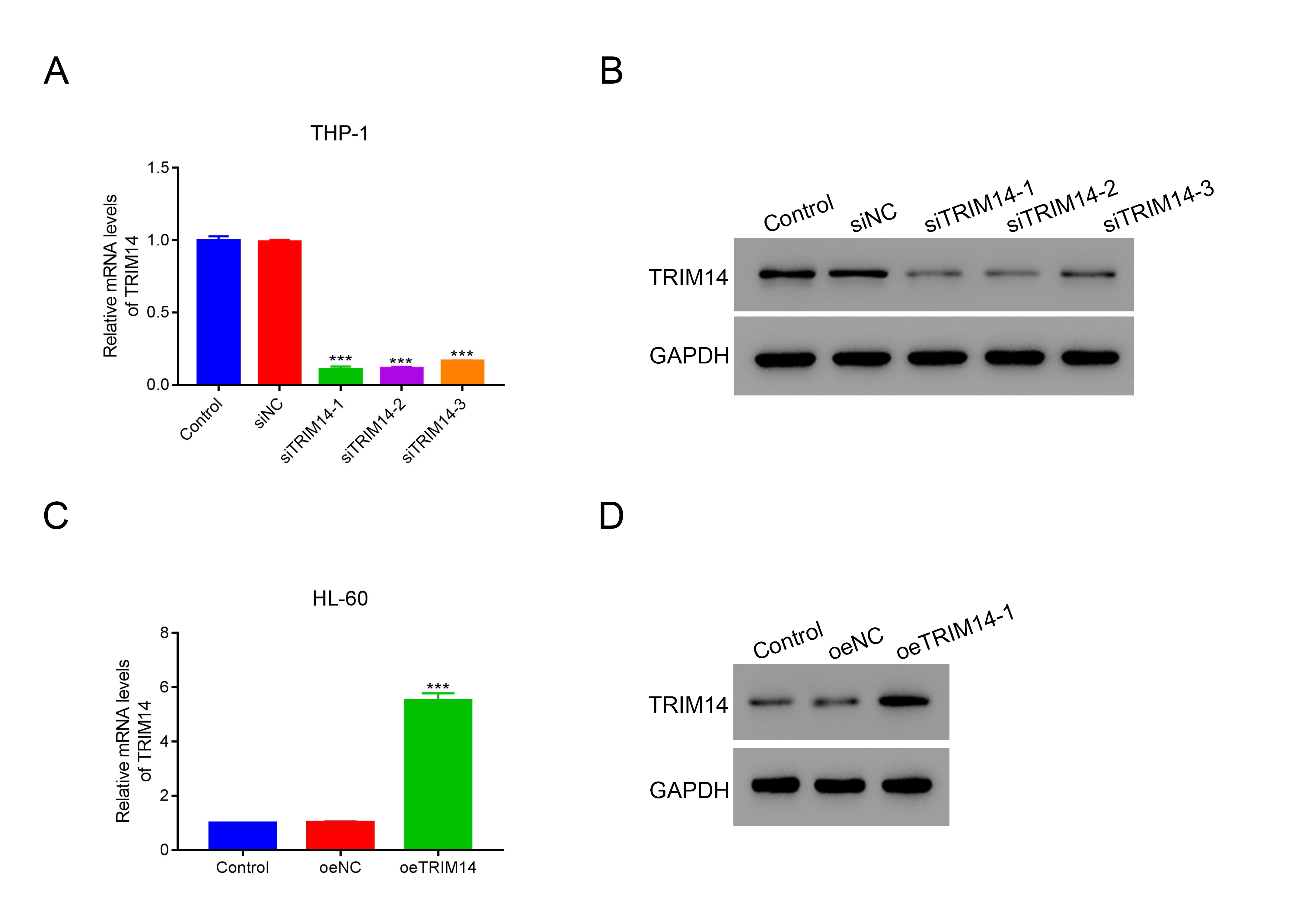

Supplement: Supplementary file 1 — Additional file 1: Fig. S1. The inhibition and overexpression of TRIM14 in THP-1cells. A–B The mRNA (A) and protein (B) levels of TRIM14 after transfecting with TRIM14 siRNAs in THP-1 cells. C–D The mRNA (C) and protein (D) levels of TRIM14 after transfecting with TRIM14 overexpressed plasmid in HL-60 cells. *** p < 0.001. [file 10020_2021_393_MOESM1_ESM.jpg]

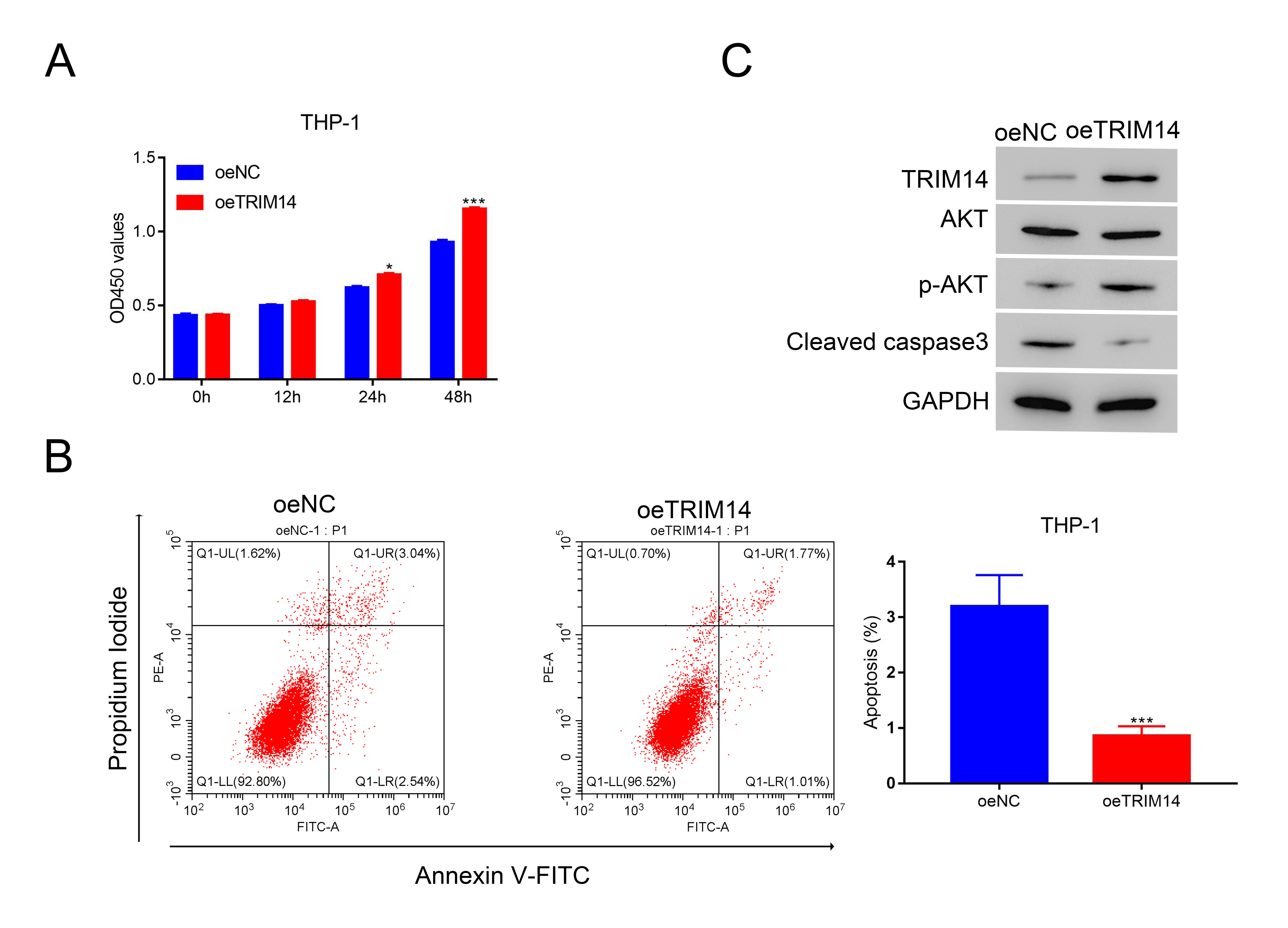

Supplement: Supplementary file 2 — Additional file 2: Fig. S2. TRIM14 overexpression promoted the proliferation and inhibited apoptosis in human THP-1 cells. A. The proliferation of THP-1 cells after transfecting with oeNC and oeTRIM14 were examined at 0, 12, 24 and 48 h. * p < 0.05 vs oeNC, *** p < 0.001 vs oeNC. B. Flow cytometer was used to examine the apoptosis of THP-1 cells after transfecting with oeNC and oeTRIM14. *** p < 0.001 vs oeNC. C. Western blot was used to examine the protein levels of TRIM14, AKT, p-AKT, cleaved caspase3 in THP-1 cells after transfecting with oeNC and oeTRIM14. [file 10020_2021_393_MOESM2_ESM.jpg]

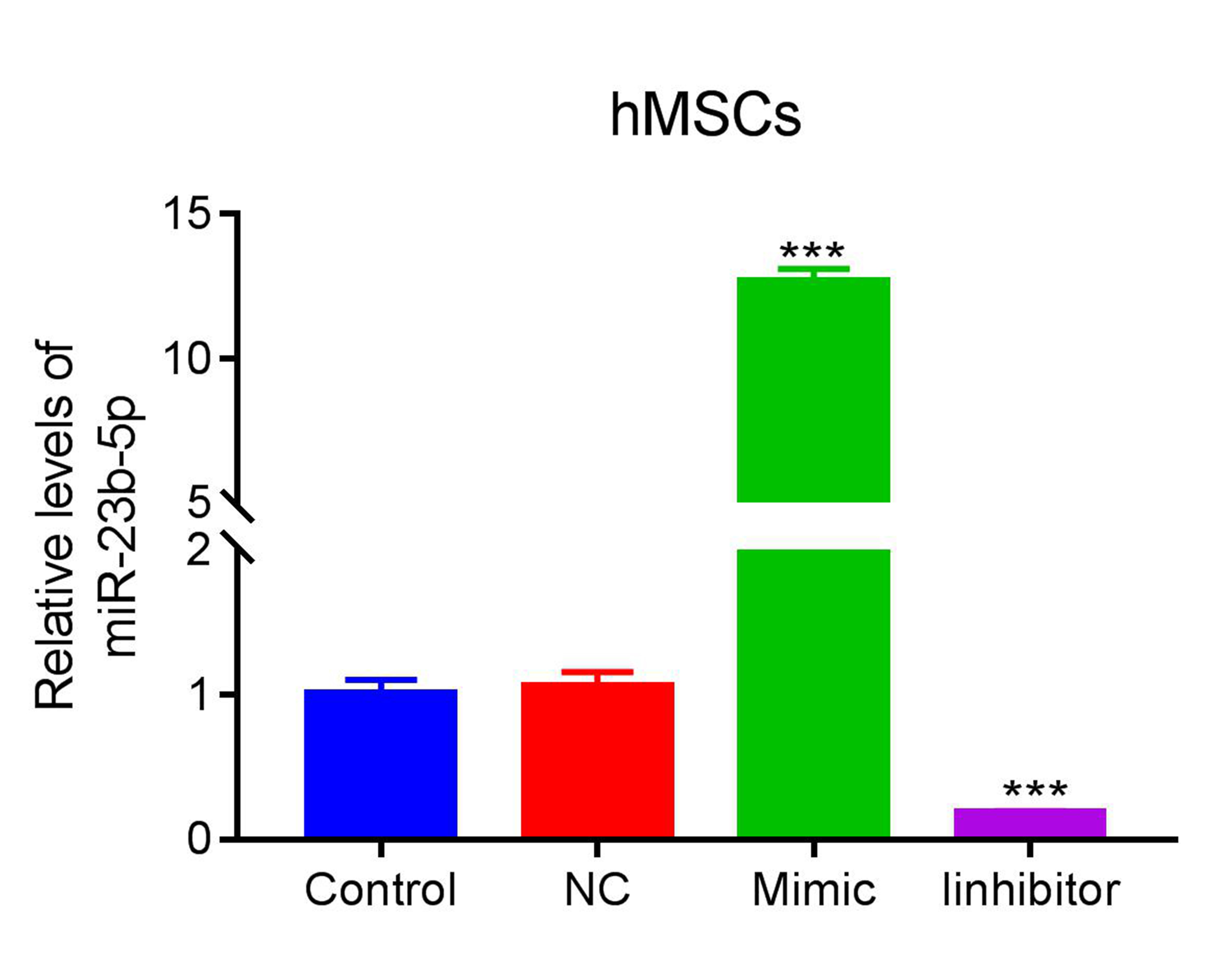

Supplement: Supplementary file 4 — Additional file 4: Fig. S4. The expression of miR-23b-5p after the application of miR-23b-5p mimic and inhibitor. *** p < 0.001. [file 10020_2021_393_MOESM4_ESM.jpg]
